# Myasthenia Gravis Induced by Immune Checkpoint Inhibitors: An Emerging Neurotoxicity in Neuro-Oncology Practice: Case Series

**DOI:** 10.3390/jcm12010130

**Published:** 2022-12-24

**Authors:** Carla Marco, Marta Simó, Montse Alemany, Carlos Casasnovas, Raúl Domínguez, Noelia Vilariño, Mariona Calvo, Juan Martín-Liberal, Jesús Brenes, Joan Sabater-Riera, Jordi Bruna, Roser Velasco

**Affiliations:** 1 Neuro-Oncology Unit, Hospital Universitari de Bellvitge-Catalan Institute of Oncology, Hospital Duran i Reynals, Institut d’Investigació Biomèdica de Bellvitge (IDIBELL), 08908 L’Hospitalet de Llobregat, Spain; 2 Neuromuscular Unit, Neurology Department, Hospital Universitari de Bellvitge, 08907 L’Hospitalet de Llobregat, Spain; 3 Department of Oncology, Catalan Institute of Oncology, Hospital Duran i Reynals, Institut d’Investigació Biomèdica de Bellvitge (IDIBELL), 08908 L’Hospitalet de Llobregat, Spain; 4 Critical Care Department, Institut d’Investigació Biomèdica de Bellvitge (IDIBELL), Hospital Universitari de Bellvitge, 08908 L’Hospitalet de Llobregat, Spain; 5 Department of Cell Biology, Physiology and Immunology, Institute of Neurosciences (UAB), 08193 Cerdanyola del Vallès, Spain

**Keywords:** myasthenia gravis, immune checkpoint inhibitors, immune-related adverse events, neurotoxicity, neuro-oncology

## Abstract

Immunotherapy with immune checkpoint inhibitors (ICIs) have been reported to induce de novo or exacerbate pre-existing Myasthenia Gravis (MG). We present a single center case series of patients who developed an immune-related myasthenia gravis (irMG) related with ICIs. We performed a retrospective chart review of the electronic medical records between 1 September 2017 and 2022. We report the clinical features, presentation forms, diagnostic workflows, general management and outcomes of six patients who received ICIs for different solid organ malignancies and developed an irMG frequently overlapping with immune-related myocarditis and/or myositis. The aim of the article is to describe the clinical features, treatment and outcomes of this challenging and potentially life-threating syndrome, comparing our data with those described in the literature. Differences between irMG and classic MG are highlighted.

## 1. Introduction 

Immune checkpoint inhibitors (ICIs) are a type of passive immunotherapy that have become part of the standard of care of many cancer types including lung, liver, pancreas, renal, breast, melanoma and lymphoma [1]. The increasing use of ICIs in patients with cancer, either in monotherapy or in combination with chemotherapy or other ICI, has led to markedly improved survival rates and longer remission periods [2]. To date, the ICIs approved by the European Medicines Agency (EMA) and Food and Drug Administration (FDA) include monoclonal antibodies targeting immune checkpoint molecules programmed cell death protein-1 (PD-1) (pembrolizumab, nivolumab, cemiplimab), or its ligand (PD-L1) (atezolizumab, avelumab, durvalumab), and cytotoxic T-lymphocyte associated protein 4 (CTLA-4) (ipilimumab, tremelimumab) [3,4]. In addition, a new ICI (relatlimab) has been recently approved against a novel target: lymphocyte-activation gene 3 (LG3) [5].

ICIs induce the immune system, blocking the co-inhibitory T-cell signals that, under normal conditions, prevent the chronic activation of the immune system. Thus, immune checkpoint inhibition can enhance the antitumor activity of T cells stimulating the destruction of the cancer cells [6]. Despite its effectiveness, several immune-related adverse events (irAEs) associated to ICI therapy have been described and any system can be affected [7]. The most commonly involved organs are the gastrointestinal tract, endocrine glands, skin and liver [8]. Neurological immune related adverse events (nrl-irAEs) are uncommon presenting an estimated overall incidence of 7.2% [9]; with severe forms occurring in up to 3% of patients receiving ICIs [10]. Nrl-irAEs include a wide spectrum of manifestations involving the entire neuroaxis, i.e., muscle, neuromuscular junction, peripheral nerve and central nervous system (CNS) [11,12,13]. Neuromuscular disorders are more frequent and have an earlier presentation than those involving the CNS [3,12]. Among neuromuscular irAEs, myasthenia gravis (MG) is the third in frequency after myositis and peripheral neuropathies including Guillain-Barré syndrome, and cranial neuropathies. However, irMG is associated with the highest morbidity and mortality rates [3,11,14]. 

Immune-related myasthenia gravis (irMG) can occur as an exacerbation of pre-existing MG or de novo in patients with no previous MG diagnosis. The clinical picture is usually characterized by progressive weakness affecting the extraocular, bulbar and limb muscles, progressing to respiratory failure in 40–65% of reported cases [13]. Some patients have mild symptoms such as ptosis while other patients may present with rapidly progressive respiratory failure with fatal outcome [15]. The presence of myositis and myocarditis overlapping irMG is a very common association. This syndrome, also named “3M triad”, is especially challenging and life-threatening [13,15,16,17]. 

An approach involving experienced neurologists in specialized multidisciplinary care teams is of great importance in the management of these patients [13,18]. In the present retrospective case-series study, we describe our experience in an oncologic center with six patients diagnosed of irMG due to ICI. Clinical, biological, radiological, electrophysiological and outcome data are described, with the objective of contributing to the knowledge of irMG and their overlaps. 

## 2. Materials and Methods

Consecutive patients with suspected ICI-related neuromuscular irAE assessed at the Neuro-Oncology Unit of Catalan Institute of Oncology-Bellvitge University Hospital (Hospitalet de Llobregat, Barcelona, Spain) on dates between 1 September 2017 and 1 September 2022, were reviewed. We included patients who met the diagnostic criteria for irMG described by the “Consensus Disease Definitions for nrl-irAEs of ICI” published on May 2021 [19]. irMG diagnosis is *definite* when the patient has symptoms, electrodiagnostic studies (EDX) and positive antibodies (Ab) (acetylcholine receptor (anti-R Ach) or Musk). A *probable* diagnosis is considered when, in the clinical context, the patient has compatible EDX or positive Ab or unequivocal clinical response with cholinesterase inhibitors. The classification includes the *possible* category when the Ab are negative (or not performed), the EDX does not show disorders of the neuromuscular junction (without irritative myopathy) but the patient has a clinical picture compatible with normal creatin kinase (CK) serum levels. We assessed the clinical severity of irMG using the Myasthenia Gravis Foundation of America (MGFA) classification. Briefly, MGFA class I is defined as ocular muscle weakness, MGFA classes II as mild weakness involving any other than ocular muscles. MGFA class III and IV are defined by moderate and severe muscle weakness, respectively. MGFA class V is defined as myasthenic crisis with respiratory failure requiring endotracheal intubation or non-invasive mechanical ventilation [20]. Additionally, the severity of irMG was classified according to the adapted the Common Terminology Criteria for Adverse Events Criteria (CTCAE) for nrl-irAEs [19]. Standard grading (severity) CTCAE scale was used for non-neurological irAE. Briefly, CTCAE displays grades 1 (mild) through 5 (death due to the AE) with unique clinical descriptions of severity [21]. Collected data from our own files included patient´s demographics and baseline characteristics (age, gender, type of cancer, ICI therapy), non-neurological associated irAEs, clinical course, serological and EDX results, treatments received, evolution and subsequent outcome. The whole of patients were assessed by neurooncologists and neurologists specialized in neuromuscular diseases, who underwent EDX studies. This retrospective study was launched after having obtained approval from the Institutional Ethics Review Board (PR309/22). Written informed consent was taken from living patients and waiver of consent from deceased patients. We excluded patients who did not meet the Guidon diagnostic criteria irMG or those for whom we did not have sufficient clinical or EDX data to be able to evaluate them properly.

## 3. Results

### 3.1. Patients 

Six patients with ICI-related MG diagnosis were included (all men); the median age at symptom onset was 74 years old (range, 65–85). No patients reported history of previous MG, thymoma, positive AntiR-Ach or anti-Musk Ab or other autoimmune diseases. All less one patient developed irMG with anti-PD1 treatment. The median follow-up from symptom onset to the last visit (or death) was 196 days [range 30–487]. Demographic, clinical, and electrodiagnostic data are summarized in Table 1.

### 3.2. Clinical Features irMG, Laboratory and Radiological Results

In our series, three (50%) patients met the MG diagnostic criteria of *definitive*, two (33%) of *probable* and one (17%) of *possible irMG* according to the recently established diagnosis immune related MG [19]. Five patients (83%) developed moderate to severe disease (MGFA III-V). Regarding the irMG onset, we can differentiate two onset patterns: those who developed symptoms early after the first ICI cycles (patients 1, 2, 3 and 6) with an early onset (median 33 days; range 10–60), and those with a delayed irMG onset (patients 4 and 5), presenting with irMG after receiving more than 30 cycles, with a median onset of 804 days [623–986 days] after first ICI dose. Noteworthy, both patients with late-onset irMG were on chronic low-dose corticosteroid treatment for previous adrenal insufficiency related to ICIs. However, no recent changes in corticosteroid doses had been done before irMG onset. 

Clinical manifestations initially were mainly cranial symptoms, including: diplopia (*n* = 5, 83%), dysphonia (*n* = 4, 67%), ptosis (*n* = 4, 67%), and dysphagia (*n* = 5, 83%). Limb weakness was present in four out of six patients (67%), three of whom had concurrent myalgia. Four patients (67%) associated both ir-myocarditis and ir-myositis. 

Blood CKs levels and troponin T were markedly increased in half of patients, with a mean of 13-fold (range 8–18) the normal value of our laboratory reference value. AntiR Ach were identified in all but one patient (83%), and titles were variable. None of the patients of the present series had anti-Musk positivity neither had Antinuclear Ab. Anti-titin antibodies were positive in two of the three (67%) patients tested.

Patient 2 had concurrent hepatitis with elevated alanine aminotransferase (ALT), aspartate aminotransferase (AST), gamma-glutamyl transferase (GGT) and coagulation disorder at irMG diagnosis. Two patients (Patient 3 and 6) had elevated ALT/AST, associated with CK peak and no other signs of hepatic failure. 

### 3.3. Electrophysiological Tests 

**Figure 1:**
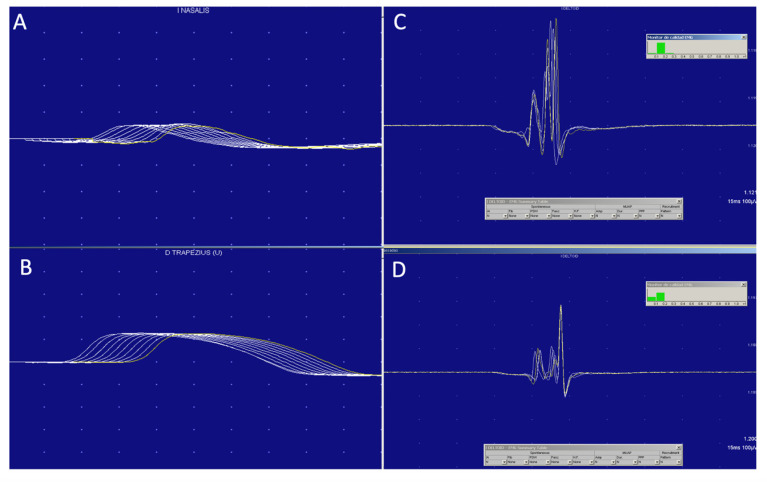
MG diagnosis is clinical, supported by antibodies and neurophysiological studies, which do not always show the classic drop in potential in 3 Hz repetitive stimulation (**A**,**B**), but, in which, the variability or the Jiggle of the motor unit (**C**,**D**) can be a very useful sign demonstrating impaired neuromuscular transmission.

Electroneuromyography including repetitive nerve stimulation (RNS) at 3 Hz and postexercise facilitation were performed in the whole series of patients. Additionally, single fiber electromyography (SFEMG) was done in four out of six patients. RNS at 3 Hz was normal in all muscles tested (*nasalis*, *trapezius* and *adductor digiti minimi*) in our six patients (Figure 1). Postexercise facilitation was also normal in all of them. Two out of four patients who underwent single fiber study had an increased jitter. Patient 3 showed an unstable motor unit potential (increase jiggle) [19,22], which can be observed in Figure 1. On needle electromyography (EMG) most patients (5/6, 83%) showed a myopathic pattern, characterized by the presence of mild spontaneous activity and myopathic recruitment (polyphasic, short-duration, or low amplitude motor unit action potential with normal or early recruitment) in proximal muscles of upper and lower extremities. The patient with an ocular form of irMG had normal EMG. 

### 3.4. Treatment and Outcomes 

ICI therapy was discontinued after irMG diagnosis in all patients, and none of them have restarted it to date. The average time from symptom onset to irMG suspicion or diagnosis and therefore treatment initiation was 28 days [4–69 days]. All but one of our patients (patient 4) required hospitalization. Corticosteroids and pyridostigmine were initially administered to all patients. In total, five patients received intravenous immunoglobulins (IGIV), in four of them administered at diagnosis concomitantly with corticoids (patient 2, 3, 4, and 6) and in one patient (patient 1) IGIV was administered due to lack of improvement. Plasma exchange (PEX) was performed in two patients due to the absence of improvement after corticosteroids and immunoglobulins. Third-line therapy with immunosuppressants like Rituximab and cyclophosphamide was considered in two patients. 

In our series, irMG presented with associated myositis and myocarditis, in the setting of the overlap syndrome called “3M triad”, in four (67%) patients. Most of them (3/4, 75%) required admission to the intensive care unit and mechanical ventilation. In two of them (patient 1 and 2) the indication for ventilation was related to respiratory failure due to the irMG and the median time between the onset of symptoms and the start of mechanical ventilation was 12 days [range 9–15]. In a third patient, mechanical ventilation was due to SARS-COV2 bilateral pneumonia (patient 3) during irMG recovery. All patients diagnosed of “3M triad” died. Their deaths were related to complications associated with the severity of their condition and not exclusively to irMG. Conversely, two patients recovered from irMG. However, very recently, patient 5 presented an irMG relapse concurrently with corticosteroids tapering. Of the two patients who survived the irMG (patient 4 and 5), both have had a partial response to the cancer and no further oncospecific treatment has been restarted to date.

Patient 3, who had concurrent ir-myositis, presented a spontaneous intramuscular bleeding in two different localizations (*brachioradialis* and *adductor magnus*) that led to a hypovolemic shock requiring blood transfusions and admission to the intensive care unit. No coagulation alteration or low platelets were detected. 

## 4. Discussion

This study presents a single center experience of a rare neurological complication due to ICI. Over a 5-year observation period, we have diagnosed six patients meeting the recently established consensus diagnostic criteria for immune-related MG in a university cancer center that covers 45% of the adult cancer population in Catalonia, Spain. The rate of ICI-treated patients who developed MG in our series is in line with previous literature, where irMG is an unusual complication, accounting for 0.5% of all irAes and 13.5% of all nrl-irAEs [3]. In recent years, some single-center case series have been published showing a cumulative incidence similar to ours. In 2018, Safa et al. published the largest case series described to date, with 14 patients from the MD Anderson Center over a 7-year observation period (2011–2018) [23], followed by Shi et al. [24] with six patients over two years (2019–2021) and Wong et al., with four patients [17]. At our center, we have observed an upward trend in the number of cases, which could be explained by the increasing use of these treatments.

Unlike classic MG, all our patients were elderly males. The predominance in this population has been previously described [23,25] and may be explainable by the target population for these treatments. Additionally, none of our patients had thymoma. In contrast to classic MG [26], thymoma does not appear to play a role in the pathogenesis of irMG [25]. Most of our patients had irMG in association with PD1 and only one with anti PDL1 agents. To date, we have not identified this type of neurotoxicity following CTLA-4 treatment. These data are similar to those published in reviews describing a higher incidence of irMG in relation to PD1/ PDL1 (86%) compared to CTLA4 alone (5%) or in combination with PD1 or PDL1 (9%) [3]. 

irMG is a complication that usually appears early after initiating ICI, within the first four cycles [23], as is the case of in most of our patients (67%). However, irMG can occur at any time during ICI treatment [27,28]. It is noteworthy that we have detected two patients with a very late onset, who started the disease after receiving more than 30 cycles of ICIs. Interestingly, both were on chronic corticosteroids treatment for adrenal insufficiency secondary to immunotherapy. However, none of our patients were on a tapering schedule, which precludes us from establishing an association with the late onset. Further research on whether this observation in causal is needed.

The clinical picture of irMG is characterized by progressive muscle weakness, with ocular, bulbar and proximal limb involvement that can progress to respiratory failure in about half of the patients [7]. Most of our patients (83%) developed moderate to severe muscle weakness (MGFA III–V) at onset, with need of mechanical ventilation in 50% of the cases. Larger case series are consistent with this feature that differentiates it from classic MG [23,29]. MG usually manifests as a milder disease and most patients fall into MGFA classes I and II at onset, with a death rate of 8% due to respiratory failure [26,30]. In Table 2 we summarize the main differences between irMG and classic MG.

In our study, we identified that 67% of our patients presented ir-myositis associated with irMG. The coexistence of myositis is frequent, and it has been reported in up to two thirds of cases in the literature [25]. In series in which a lower incidence was reported, underdiagnosis has been suggested [23]. It is noteworthy that two thirds of our patients were diagnosed with ICI-related myocarditis, which is higher than expected (estimated prevalence varying from 13% [29] to 31% [25]). Overall, the ratio of patients with the “3M triad” in our series (67%) is also higher than that described in the literature, with a reported estimated prevalence of 8% [23]. A higher clinical suspicion and an active search for the concurrent syndromes could explain our results. Regarding the EDX results, half of our patients showed findings compatible with neuromuscular junction impairment with pathological jitter to jiggle, but none had RNS with pathological decrement of amplitude. These studies are in line with previously published data describing findings compatible with neuromuscular junction involvement in up to 50% rate of the cases [7,13]. Remarkably, all the patients with generalized myasthenia in our series displayed a myopathic pattern with mild spontaneous activity in proximal muscles, which is a finding also described in classic MG [31,32]. These findings can be difficult to differentiate from myositis, and for this reason we have not relied solely on EDX findings to diagnose an immune-mediated myopathy. We have taken into consideration the clinical picture, CK levels and the presence of moderate spontaneous activity together with a myopathic pattern. A limitation this report is the absence of muscle biopsy or muscle image to confirm the diagnosis. Importantly, the diagnostic criteria reported by Guidon et al., rely on EDX findings for diagnosis, but they are not essential to classify MG as *probable*. [19]. As in classic MG, EDX findings are not required to confirm the diagnosis [30]. This facilitates the diagnosis of irMG but may lead to a misdiagnosis of ir-myositis in patients with cranial or respiratory involvement without elevated CK [15]. The combination of these syndromes (irMG and ir-myositis) confers the patient a different prognosis than presenting them separately, highlighting importance of investigating their concomitant presence [25]. 

In a case of isolated ocular symptoms, it is important to make a broad differential diagnosis (thyroid eye disease, ocular myositis, myasthenia gravis, tumor or vascular compression, etc.). In our patient (patient 4), the acute onset without pain, the clinical fluctuation, the fatigue and the normal brain MRI were the key to the diagnosis.

Most of our patients (83%) had positive AChR Ab, which is a slightly higher rate than that published in previous cases series or reviews [7,23], where AChR Ab positivity varied, ranging from 50% [25] to 66.7% [29]. None of our patients had anti-MUSK antibodies. Its positivity has been reported anecdotally in irMG [25]. 

Therapeutic management included ICI withdrawal in all cases; initiation of acetylcholinesterase inhibitors; early use of corticosteroids; and IVIG and/or PEX in case of non-response or worsening [33,34,35]. A very recent study showed that patients with irMG may benefit from initial therapy with IVIG or PEX regardless of initial severity [23], which was applied in the most recently diagnosed patients of ours series. Unlike the idiopathic form, irMG can be monophasic [36], so additional corticosteroid sparing agents may not be always necessary. Refractory cases have been reported and the use of mycophenolate mophetil or rituximab has been required [13]. 

IrMG can occur associated with other neurological or non-neurological irAEs. As it has been widely described the association of irMG with other irAEs are highly common [23,37]. Among our patients, myocarditis and myositis were the most frequently observed (67%), followed by hepatitis (17%). Importantly, the elevation of ALT/AST without elevation of GGT should be interpreted carefully [35] as it may be elevated due to rhabdomyolysis and not to hepatitis. Furthermore, irMG relapses could be associated to the management with corticosteroids in other irAEs. In our series, two patients developed irMG and one patient relapsed in the setting of corticosteroids tapering. 

Unfortunately, four out of six (66.7%) patients had a fatal outcome in our series, reaching with 100% mortality rate in patients with the 3M triad. It is noteworthy that not all deaths were directly related to respiratory insufficiency, some of there were related to other systemic complications like SARS-CoV-2 or faecaloid peritonitis (see outcome). Our results contrast with the outcome in other series where partial or full recovery of irMG was observed in 70% of the cases that received adequate and prompt treatment [3]. However, in the literature, mortality due to irMG is much higher than expected in classic MG (28–30% vs. 6%), as a consequence of respiratory failure [13]. The coexistence of other irAES increases the risk of mortality, being 35% in patients with ir-myopathy and 60% in cases with ir-myocarditis [25]. irMG presenting with both myocarditis and myositis is known to carry the highest death rate (5/8, 62.5%) [37].

Some limitations should be acknowledged from the present study. The small sample size and the retrospective nature of the study design limit the reliability of our results. Furthermore, one of the six patients was categorized as possible irMG. However, all of them fulfilled the recently proposed diagnostic criteria for irMG. However, specialized and multidisciplinary evaluation limiting bias (neurooncologist and neurologist) and a long follow-up must be highlighted.

## 5. Conclusions

ICI-related MG is a rare but often life-threatening complication, especially in those patients presenting with the 3M triad. The frequency of irMG will likely increase as the use of ICI becomes more common. Importantly, some differences with classic MG syndromes regarding clinical presentation, management and outcome have been observed, highlighting the need for detailed descriptions of this challenging entity. Clinicians should be aware of this complication having a high index of clinical suspicion and they should perform a prompt and thorough investigation, including an active search for the most frequently associated syndromes: myositis and myocarditis. Early involvement of experienced neurologists in the oncologic multidisciplinary team, initial discontinuation of ICI and treatment with corticosteroids and immunomodulators are key aspects in the management of this neurological complication.

## Figures and Tables

**Table 1:** Characteristics of patients with irMG.

	Patient 1	Patient 2	Patient 3	Patient 4	Patient 5	Patient 6
Age (y)/gender	77 y/man	78 y/man	70 y/man	65 y/man	69 y/man	85 y/man
Neoplasm	NSCLC	Prostate	Melanoma	Hepatocarcinoma	NSCLC	NSCLC
ICI	Spartalizumab	Pembrolizumab	Nivolumab	Tislezizumab	Pembrolizumab	Durvalumab
Days after first ICI dose	60	19	15	623	986	37
Cycles of ICI	3	1	2	31	35	4
Year irMG diagnosis	2017	2019	2020	2021	2021	2022
Symptoms	Diplopia, ptosis	Diplopia, ptosis	Diplopia	Diplopia, ptosis	Diplopia	Ptosis
	Dysphonia		Dysphonia		Dysphonia	Dysphonia
		Dysphagia	Dysphagia		Dysphagia	Dysphagia
	Head drop					
	Limb weakness		Limb weakness		Limb weakness	Limb weakness
	Myalgia		Myalgia		Myalgia	
	Dyspnea	Dyspnea			Dyspnea	
	Chest pain					
Creatine- kinase	×12	×8	×18	N	N	N
T Troponin	×158	×161	×76	N	N	×62
MG Auto Abs (N < 0.45)	+[6,14]	+[6,86]	+ [0,68]	-	+ [1,66]	+ [>20]
EMG	Myopathic	Myopathic	Myopathic	Normal	Myopathic	Myopathic
RNS 3 Hz/Jitter	N/NA	N/NA	N/Jiggle	N/NA	N/Pathological	N/Pathological
MGFA	IVB	V	V	I	IIIA	IIIB
IrMG CTCAE	G4	G4	G4	G2	G3	G3
Myocarditis	Yes	Yes	Yes	No	No	Yes
Myositis	Yes	Yes	Yes	No	No	Yes
grade CTCAE	G4	G4	G4			G4
Concurrent other irAEs	No	Hepatitis G3	No	No	No	No
Previous irAEs	No	No	No	Endocrine G2	Endocrine G2	No
Treatment						
Corticoids	Yes	Yes	Yes	Yes	Yes	Yes
IgEV/PEX	Yes/Yes	Yes/Yes	Yes/No	No/No	Yes/No	Yes/No
Other	-	Rituximab	CFM	-	-	-
Mechanical ventilation	Yes	Yes	Yes	No	No	No
Follow-up in days	60	133	53	467	434	30
Final outcome	Death	Death	Death	Full recovery	Relapse irMG	Death

AntiR-Ach: anti receptor acetylcholine; AutoAbs: auto antibodies; CFM: Ciclofosfamide; CMV: cytomegalovirus; CTCAE: Common Terminology Criteria for Adverse Events; ECG: electrocardiogram; EIT: endotracheal intubation; EMG: electromyography; G: grade; ICI: immune checkpoint inhibitor; IgEV: immunoglobulins endovenous; IrAEs: immune related adverse events; irMG: immune related myasthenia gravis; MG: myasthenia gravis; MGFA: Myasthenia Gravis Foundation of America (clinical classification); MRI: magnetic resonance image; MUP: motor unite potential; N: normal; NA: not available; NIV: non-invasive ventilation; NSCLC: non-small cells lung cancer; PEX: plasma exchange; PD1: programmed death; RNS: repetitive nerve stimulation; SARS-COV2: severe acute respiratory syndrome COVID 2; y: years.

**Table 2:** Differences between immune-related and idiopathic forms of MG.

Feature	irMG	Classic MG
Population	Elderly Males	Woman < 30 Years Man > 70 Years
Isolated ocular presentation	−	+/−
Associated with thymoma	−	+
Associated with myositis +/− myocarditis	+	−
Anti AchR ^1^ Antibodies	+	++
Musk Antibodies	−	+
Abnormal repetitive nerve stimulation	+/−	+
Mortality due to MG	+	−

^1^ AchR:Acetylcholine Receptor; MG: Myasthenia Gravis.

## Data Availability

Not applicable.
